# The Circulation

**Published:** 1869-09

**Authors:** N. M. Burkholder


### ARTICLE II.

### *The Circulation.*

By N. M. Burkholder, D.D.S.

The blood is the life of the body. The circulation of it throughout the system is necessary to life, to the performance of the functions of nutrition and secretion, in that it gives to all the tissues a constantly renewed supply of the materials which they severally require in order to growth—development, and also provides through its channels a means of escape for those excrementitial matters, which are the constant result of the decomposing tissues. The different structures require different materials. The muscle, for instance, wants fibrine, the nerve, fatty matter, the bone, gelatin and earthy salts, the nutrition of the milk cells during lactation separates albumenous, fatty and saccharine substances and so on, and we observe that the blood when returning from the different organs through which it has been transmitted, has undergone very different changes, according to the requirement of the organ or tissue which each current has supplied, and if the same portion of the blood were being constantly transmitted to each organ, on its return, its composition would speedily undergo a change, which would render it useless, but by the general circulation or commingling of all the smaller circulations, before the blood is again propelled by the central organ outward to the tissues, this change is prevented and the supply of nutritive material, which the blood has been robbed of, is again restored by the transformation of albumen by the aid of the lymphatics and, mainly, by the continual supply of chyle and oxygen.

Again the living body is subject to perpetual change. Death commences the first moment of our existence. Chemical laws rule without intermission, and all the exhibitions of life are but the result of chemical or chemico-vital change. An innumerable play of affinities surrounds us, theirs is the grand ruling law of the universe, unchangeable and unremitting, without these laws we have no *growth*, no *decomposition*, no *change*. We are but a compound of elements, formed in accordance with these laws, presided over, however, by an immaterial Principle, the essential nature of whose connection with matter, *who is able to understand?* One of the primitive elements which help to make up the tissues is oxygen, another carbon. Oxygen is being continually eliminated in combination with other elements, from the system, chiefly as a vehicle for the escape of carbon in the form of carbonic acid gas, the retention of which is incompatible with the life of the nervous system. Therefore a constant supply of fresh oxygen is required for the life of the animal.

The lungs is an apparatus in the air cells of which the oxygen of the atmosphere meets with the blood, which absorbs it and throws off carbonic acid, which the ablest physiologists of the present day consider a result of the decomposition of animal tissues, and not, as has been supposed, a direct result of the oxidation of carbon. Here then is another of the great purposes of the circulation, in the conveyance of oxygen directly to the tissues, to the ultimate germs of life, for their appropriation and for their development and the bearing away of carbonic acid, resulting from the breaking down of formed material or the decomposition of the body, which is now excrementitial. All the excrementitious matters resulting from the decomposition are washed away in this wonderful current, some to be eliminated through the liver, some by the kidneys, some by the lungs and some passing directly through the skin, each by its appropriate gate and often performing functions on the way with relation to other vital processes, which (functions) are essential to life.

And here I may briefly notice the natural eye appearance of the blood. That current which having passed through the capillaries of the lungs and there parted with its carbonic acid and freighted itself with oxygen and passing into the left side of the heart is thrown out to feed the tissues, is found to have a bright scarlet color and is called *aerated*, oxygenated or arterial blood, after passing through the great capillary system of vessels, it is found in the veins and right side of the heart, beyond, to have a dark purple (and almost black) hue, and this is characteristic of impoverished blood, an absence of oxygen and a heavy freight of carbonic acid. These are mere facts, and when we are asked how the oxygen combines with the globules, albumen, salts, &c., we have only to say *it is not known.*

In the higher forms of animal life we find an apparatus for the circulation, which, in its nature, is as complicated as it is wonderful. In the vegetable world and by consequence in the lower classes of animal life, which possess but little higher expressions of vitality, we find a circulation of the most simple and rudimentary form. The nature of the force by which the circulation is carried on in any of the higher plants, can be easily learned upon minute examination. Those who have studied these things tell us there is an ascending and a descending sap. This ascending sap consists chiefly of water which holds various substances in solution and has been absorbed or taken up by the soft sponge-like rootlets. The laws of capillary attraction explain this movement, in part, as its *vis a tergo.* We see this power of forcing up a column of sap very readily when we cut across the stem of a vine or plant in which the sap rises rapidly and which is in full bloom or leaf. This powerful " vis a tergo" has been estimated at from 15 to 40 lbs. to the square inch, but there is also a condition of things on the reverse side, which not only aids, but which is the chief force upon which the circulation depends, and this is the nutritive force generated by the chemico-vital changes taking place in the performance of the nutritive function. Light and heat—physical conditions are necessary—they stimulate the exhaling process by which the watery portions of the ascending sap are thrown off. Darkness and cold check this exhalation and of course check the absorption at the lower extremity, these with others are modifying influences only.

While we might compare the fluid in question to the chyle, the next in hand, the descending sap, may be strictly compared to the blood of animals. The carbon which has been taken in by the cells of the leaves, in combination with oxygen (carbonic acid) forms with the remnant of the ascending fluid after exhalation has taken place, a gummy matter, which is in a state ready to be appropriated by and developed into solid structure and is conveyed by a network of vessels to the exterior and chiefly downwards to the roots.

There is an affinity between certain particles of the nutrient fluid and the solid matter through which it flows. There is then a constant attraction of these particles towards the walls of the vessels, these particles satisfy the body attracting them, affinity is accordingly lost, they pass on being pressed on by the force which attracts particles ever behind, and thus the movement may be onward or backward, regular or irregular in its course, very much influenced to be sure by heat, cold, exercise etc. In all the capillary network the flow is maintained in this manner by what we choose to designate *osmotic action.* If we personify the nutritive particles we have a grand market, in which the order is entirely reversed, for instead of person seeking food, food seeks and finds the person.

In vegetable life we have no need of a central propelling organ for the reason that all the fluid seems to be appropriated by the tissues through which it has circulated; neither is there necessity for much oxygen. In some of the lower classes of animals, as also in one stage of man's primary or embryonic state, we have a circulation very nearly alike to this. Not forgetting in these comparisons the one wide distinction between the vegetable and animal circulation, viz: That the function of the circulation in the vegetable world is for growth alone, while in the animal it has the additional office, as we have already stated, of conveying to the exterior the results of a constantly decomposing tissue. Now, in the lowest forms of animal life, we have no blood vessels, just as we have no sap vessels in the sea weeds, the whole substance possessing nearly the same degree of absorption is nourished by direct absorption, as also in the first stage of the embryonic state of man. Coming a little higher in the scale of animal life, we find vessels of circulation, the fluid governed in its movement by the changes resulting from the performance of the functions of growth, secretion, &c., as already mentioned.

In other words to express the law:—

Growth—Development is the power inherent in the germ, it is this which gives it its individuality, and granting the possession of this power, we necessarily acknowledge and include the truth of the following theory: "Wherever blood is necessary, a flow is set up by laws of a vital or chemico-vital nature."

Now, in the human embryo, the primitive germinal granule, possessed of an individuality which nothing can change, this power of development under certain conditions, the basis of all vital influence, forms its vessels, it is said, by the aggregation of cells, in which the first blood seems to be formed. These vessels are first formed in a membranous expansion or covering which surrounds what we call the yolk (of the egg) which serves as a sort of temporary stomach, and capillaries are first formed necessarily, being all this condition requires, fine and almost imperceptible lines of red shooting across this membrane. After a short time larger trunks appear by the union of the smaller. The first direction of the flow (visible) is to the germinal centres, where the little organism is working away silently yet continuously toward her wonderful destiny.

Higher and higher in the scale of organic life, rises this little wonder and the young heart has not yet become muscular, when the blood begins to flow in by a union of the vessels, towards where we afterwards find it located. We now see that the heart is the child of the blood. The old theory that the heart was the sole supporter of the circulation has long since exploded and we know that the heart is only developed when needed by the organism. In some of the lower classes of animal life, the Hydra for instance, we have the functions performed by a heart, lungs and stomach, all performed by a single hollow sack higher up in the reptile we have a heart into one common ventricle of which all the blood returns and is again sent forth. In the fish we have a single or venous heart, the blood after being aerated in the gills not returning to the heart to be thrown out to the systemic capillaries, but passing immediately from the gills into the capillary system, another indisputable proof of the power of osmotic action. This is purely a single or venous circulation.—Let us stop tracing the development of the human heart and look at it in its highest possible form, under the law which determines the limits of all living objects. The human heart secures a double circulation and may be said to have two distinct parts, viz: a systemic or arterial heart and a respiratory or venous heart, the former its left and the latter its right side, each part has a receiving cavity called the auricle and an impelling cavity called the ventricle. These two sides (except in fœtal life) are separated from one another. The position of the heart is between the fifth and sixth ribs, impulse felt two inches to the left of the sternum, hangs pointing to the left, resting upon the tendinous portion of the diaphragm, the base, or longitudinal diameter, passing upwards and to the right and the point downwards and to the left, between the two layers of the pleura and enclosed in a fibro-serous membrane called the pericardium. Length five inches, breadth three, thickness two inches. Weight in males ten to twelve ounces, females eight to ten ounces. The doorway between the auricles and ventricles is guarded by valves to prevent regurgitation, those between the right auricle and ventricle called the tri-cuspid valve from their shape, those between the left auricle and ventricle, the mitral valves for the same reason. At the neck of the aorta and pulmonary artery we also have valves, called the semi-lunar. Two large venous trunks under the right auricle, one descending, the other ascending called the superior and inferior vena cava respectively. The former enters in such a direction as to pour its current directly into the auriculo-ventricular opening, the latter, however, pours its current into the posterior inferior portion of the auricle, against the septum between the two auricles at the point, the "fossa ovalis," at which, in the fœtus, it passed through a foramen, the "foramen ovale" into the left auricle, is mingled with the other current and passes into the right ventricle, from which cavity the black carbonated blood, is impelled through the pulmonary artery to the lungs and in its passage through the capillaries of which, having thrown off its carbonic acid and being loaded with oxygen, it returns by the pulmonary veins to the left auricle, thence passing into the left ventricle, is impelled through the aorta and its branches outward on its life giving mission. These are all the vessels of this organ except those which nourish its own structure called the coronary vessels. The ductus arteriosus we shall notice when we come to speak of fœtal circulation.

In regard to the structure of the blood vessels, 1st, The arteries have an external condensed layer of alveolar tissue. (2) Elastic tissue. (3) Circular arrangement of the muscular around the tube. (4) Brittle, polished, thin membrane pierced with minute foramina. (5) Tesselated epithelium. In the large arteries the elastic coat predominates, further out the muscular coat gets the advantage of the elastic, and in the capillaries, that great network of minute canals, the thin semi elastic basement membrane is its only investment and is extremely thin, having lost all the others. The veins have more muscular coat than elastic, they have valves opening towards the heart strengthened by the smallest quantity of fibrous tissue; veins in their natural structure will sustain a pressure which will rupture the arteries. The force of the circulation in the aorta is 4 lbs. and 4 ℥, and said to be about 1-12th that force in the veins. The relative rapidity of its current compared to the veins is as 12 to 8. The blood is not forced to the arterial terminations by the simple force of the heart by any means, but the elastic coat of the aorta first and on being distended by the column of driven blood, recoils and contracting upon its own volume, propels the current onward, in regular wave like currents to the arterial ramifications when the osmotic action of the capillary system assumes partial if not entire control of its movements, and in which *mysterious vale* the chemico-vital processes nutrition, secretion, etc. are carried on,

Let us return to the question of osmosis and examine the point somewhat by way of proof, promising to be very brief.

We know that the blood in the capillaries of the lungs gives out carbonic acid and absorbs oxygen, and that in the systemic arteries and capillaries it gives out to the tissues part of its oxygen and takes up the carbonic acid. Now if either of these changes be stopped, a complete stagnation of the blood will very soon take place, a damming up of the blood in the arteries, and, per necessity, a laboring action of the heart. If then oxygen be readmitted the movement will be renewed, provided that this suspension has not been too long continued. We may undoubtedly explain this by the laws of affinity, for the blood containing oxygen which the tissue requires, must possess greater affinity for the tissues than the venous blood which has given up its oxygen and hence we find the venous being continually pushed on ahead as the attraction of the arterial presses on it from behind, and once this arterial current fails to receive oxygen in the lungs it no longer possesses the affinity necessary for the production of this movement, hence the stagnation, caused by an absence of the osmotic action of the capillaries.—Another proof is incontestibly shown in the distension of the right side of the heart and large communicating vessels and partial vacuity of the reverse or arterial side, after death in certain cases, caused by the drain of the blood after saumatic death by osmotic force through the capillary system and which has entirely ceased at the time of rigor mortis. The reverse phenomena would most surely obtain if it were true that the force exerted upon the blood by the heart was sufficient to explain the entire phenomena of the circulation. The complexity of affinities is the explanation for the complexity of movement in the capillaries.

*To be Continued.*

---

### ARTICLE III.

### *Southern Dental Association.*

